# Association between diverticular disease and colorectal cancer: a bidirectional mendelian randomization study

**DOI:** 10.1186/s12885-023-10606-x

**Published:** 2023-02-10

**Authors:** Yanxi Zhang, Han Zhang, Jinghan Zhu, Yazhou He, Peng Wang, Doudou Li, Xiaozhuan Liu, Wen Jin, Junxi Zhang, Chuan Xu, Zengli Yu, Xin Zhao, Lingling Cui

**Affiliations:** 1 The First Affiliated Hospital, Zhejiang University School of Medicine, Hangzhou, China; 2 College of Public Health, Zhengzhou University, 450001 Zhengzhou, Henan China; 3 The Second School of Clinical Medicine, Southern Medical University, Guangzhou, Guangdong China; 4 Department of oncology, West China School of Public Health and West China Fourth Hospital, Sichuan University, Chengdu, China; 5 Henan Key Laboratory of Tumor Epidemiology and State Key Laboratory of Esophageal Cancer Prevention & Treatment, Zhengzhou University, Zhengzhou, China; 6 Henan Provincial People’s Hospital, Zhengzhou University, Zhengzhou, China; 7 NHC Key Laboratory of Birth Defects Prevention & Henan Key Laboratory of Population Defects Prevention, Zhengzhou, China; 8 Department of Oncology, Sichuan Provincial People’s Hospital, Sichuan Academy of Medical Sciences, University of Electronic Science and Technology of China, Chengdu, China; 9 The Third Affiliated Hospital, Zhengzhou University, Zhengzhou, China

**Keywords:** Colorectal cancer, Diverticular disease, Mendelian randomization

## Abstract

**Background:**

Diverticular disease has been inconsistently associated with colorectal cancer risk. We conducted a bidirectional Mendelian randomization study to assess this association.

**Methods:**

Forty-three and seventy single-nucleotide polymorphisms associated with diverticular disease and colorectal cancer at the genome-wide significance level (*p* < 5 × 10^− 8^) were selected as instrumental variables from large-scale genome-wide association studies of European descent, respectively. Summary-level data for colon cancer, rectum cancer, and colorectal cancer were obtained from genome-wide association analyses of the FinnGen consortium and the UK Biobank study. Summary-level data for diverticular disease was derived from a genome-wide association study conducted in the UK Biobank population. The random effect inverse-variance weighted Mendelian randomization approach was used as the primary method and MR-Egger, weighted-median, and MR-PRESSO approaches were conducted as sensitivity analyses.

**Results:**

Genetically determined diverticular disease was associated with a higher risk of colorectal cancer (beta = 0.441, 95%CI: 0.081–0.801, *P* = 0.016) in the FinnGen population, but the association was not found in the UK Biobank (beta = 0.208, 95%CI: -0.291,0.532, *P* = 0.207). The positive association remained consistent direction in the three sensitivity analyses. In the stratified analysis in the FinnGen consortium, an association was found to exist between genetically predicted diverticular disease and colon cancer (beta = 0.489, 95%CI: 0.020–0.959, *P* = 0.041), rather than rectum cancer (beta = 0.328, 95%CI: -0.119-0.775, *P* = 0.151). Besides, we found a slight association between colorectal cancer and diverticular disease (beta = 0.007, 95%CI: 0.004–0.010, *P* < 0.001) when using colorectal cancer as exposome and diverticular disease as outcome. However, there is a large sample overlap in this step of analysis.

**Conclusion:**

This Mendelian randomization study suggests that diverticular disease may be a possible risk factor for colorectal cancer and colon cancer rather than rectum cancer in the FinnGen population.

**Supplementary Information:**

The online version contains supplementary material available at 10.1186/s12885-023-10606-x.

## Introduction

Diverticular disease is a common digestive tract disease and has been associated with colorectal cancer risk in epidemiological studies [1]. A cohort study including 445,456 Danish adults found that patients with diverticular disease had a 120% higher risk of colon cancer compared to those without diverticulitis after an 18-year follow-up period [2]. This positive association was also revealed in several other observational studies [3–5]. However, findings based on two large databases negated the positive long-term association between diverticular disease and colorectal cancer and proposed that the increased risk of colorectal cancer in the first year after the diagnosis of diverticular disease was likely caused by surveillance bias and misclassification [6, 7].

Some certain embedded limitations (e.g., residual confounding) of observational studies impede the inference of causal associations between exposures and outcomes, and randomized controlled trials are not suitable for studying the association between diverticular disease and colorectal cancer. Therefore, it seems that Mendelian randomization (MR) analysis is a more feasible study strategy. Using genetic variants as instrumental variables for exposure (e.g., diverticular disease), MR analysis can strengthen the causal inference in exposure-outcome associations by minimizing residual confounding and reverse causality [8]. Here, we conducted a bidirectional MR study to examine the potential causal association between diverticular disease and colorectal cancer.

## Materials and methods

### Study design

To evaluate the association between diverticular disease and colorectal cancer, we performed a bidirectional MR study. Instrumental variables of diverticular disease and colorectal cancer were derived from large-scale genome-wide association studies (GWAS). Summary-level data on colorectal cancer were obtained from the FinnGen and UK Biobank, while that for diverticular disease was derived from a large-scale genome-wide association study. The random effects inverse-variance weighted MR approach was applied as the primary method, along with weighted median, MR-Egger, MR-PRESSO methods as sensitivity analyses. In addition, stratified analysis by site-specific cancer was performed to evaluate the association between diverticular disease and colon/rectum cancer risk with the summary-level data obtained from the FinnGen and UK Biobank.

### Genetic instrument selection

Fifty-one single nucleotide polymorphisms (SNPs) associated with diverticular disease at the genome-wide significance threshold (*p* < 5 × 10^− 8^) were identified from the discovery stage of a genome-wide association study, which included 31,964 cases and 419,135 controls coming from the UK Biobank and were performed based on the Version 3 imputed genotypes [9]. By using BOLT-LMM v2.34, a linear mixed model was performed to evaluate the genome-wide association with adjustment for the effects of population structure and individual relatedness [10]. At the replication stage, a combined European sample of 3,893 cases and 2,829 controls was included. Details of the replication sample were described elsewhere [9]. Three SNPs (rs12942267, rs3752946, and rs12041565) with the opposite effect at the replication stage were excluded. Linkage disequilibrium (LD) (*r*^*2*^ > 0.01 or clump distance < 10 000 kb) among the remaining 48 SNPs was estimated based on the 1000 Genomes European reference panel [11]. Five SNPs (rs111316530, rs139760870, rs147496465, rs575909118, and rs72221075) in linkage disequilibrium were removed, leaving 43 SNPs as instrument variables. In addition, we calculated F-statistics for each SNP to exclude weak instrumental variables (F < 10), and no weak instrumental variable was found. These SNPs can explain a 1.26% variance for diverticular disease (Supplementary Table 1). Due to the sample overlap between the exposure and outcome in the UK Biobank, we also employed the beta and se from the replication stage [12] of the original GWAS for diverticular disease to validate our primary findings in the UK Biobank (Supplementary Table 2).

SNPs of colorectal cancer at the genome-wide association (*p* < 5 × 10^− 8^) were obtained from a combined meta-analysis of GWASs with a sample of 125,478 individuals [13]. By using a logistic regression model and three stages of meta-analyses, researchers identified 95 variants that were associated with colorectal cancer [13], and 78 of them reached genome-wide significance (*p* < 5 × 10^− 8^). As mentioned above, we evaluated LD among these SNPs and eight SNPs were excluded due to LD (*r*^*2*^ > 0.01). We also determined the weak instruments by calculating F-statistics for each SNP, and no weak instruments was detected. The variance of colorectal cancer can be explained by the used instruments was 16.97%. (Supplementary Table 3)

### Data sources for outcomes

Summary-level data on associations of diverticular disease-associated SNPs with colon cancer, rectum cancer, and colorectal cancer were obtained from the FinnGen consortium [14] and the UK Biobank study (https://pan.ukbb.broadinstitute.org/). Detailed information on these two consortiums is presented in Supplementary Table 4. Seven SNPs (rs2056544, rs505922, rs6001870, rs60869342, rs62125298, rs7624168, and rs7990) had no effect estimates on these three cancers in the FinnGen consortium study.

The effect estimates of colorectal cancer-associated SNPs on diverticular disease were derived from a publicly available meta-analysis of GWASs, which was conducted among 31,964 cases and 419,135 controls in the UK Biobank [9]. The data download link was provided in Supplementary Table 4.

Ethics committee approval and participant informed consent were obtained by each study. All the estimates used in the MR analyses were displayed in Supplementary Tables 5–14.

### Statistical analysis

The diverticular disease was used as exposure and colorectal cancer was employed as the outcome in the primary MR analysis, and the two roles are swapped when investigating the effect of colorectal cancer on diverticular disease. The random effect inverse-variance weighted MR method was used as the primary method, and MR estimates were performed in beta values because the exposure and outcome are all binary variables [15]. Three main assumption should be considered when conducting MR analysis [16]: (1) instrumental variables are strongly correlated with exposures of interest; (2) instruments are not related to the potential confounders; (3) the selected genetic variants should affect the outcome only via the exposures of interest. Several sensitivity analyses, including the weighted median [17], MR-Egger [18], and MR-PRESSO [19] methods, were conducted to examine the consistency of results and to detect horizontal pleiotropy. The weighted median method can provide consistent causal estimates if more than half of the weight comes from valid instruments [17]. MR-Egger regression can detect horizontal pleiotropy by its intercept and generate an estimate with adjustment for pleiotropic effects; however, it has less statistical power [18]. The MR-PRESSO method can detect SNPs that are outliers and provide a causal estimate after the removal of these outliers [19]. The embedded distortion test can detect the difference between estimates before and after the removal of outliers [19]. Besides, Cochrane’s Q test was used to assess the heterogeneity among estimates of SNPs in one analysis. All tests were two-sided and performed using the “TwoSampleMR” (version:0.5.6) [20] and “MR-PRESSO” (version: 1.0) [19] packages in the R software (version 4.1.3). This MR study was reported according to the STROBE-MR checklist [21].

## Results

**Fig. 1:**
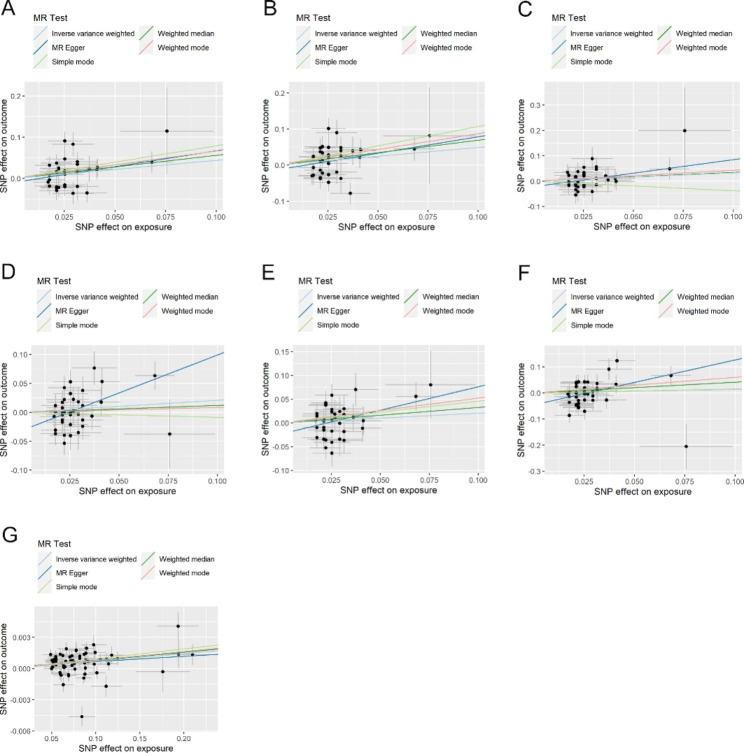
Scatter plots of Mendelian randomization analysis. A, scatter plot of Mendelian randomization analysis for diverticular disease on colorectal cancer in the FinnGen Biobank. B, scatter plot of Mendelian randomization analysis for diverticular disease on colon cancer in the FinnGen Biobank. C, scatter plot of Mendelian randomization analysis for diverticular disease on rectum cancer in the FinnGen Biobank. D, scatter plot of Mendelian randomization analysis for diverticular disease on colorectal cancer in the UK Biobank. E, scatter plot of Mendelian randomization analysis for diverticular disease on colon cancer in the UK Biobank. F, scatter plot of Mendelian randomization analysis for diverticular disease on rectum cancer in the UK Biobank. G, scatter plot of Mendelian randomization analysis for colorectal cancer on diverticular disease

Genetic predisposition to diverticular disease was associated with increased risks of colorectal cancer (beta = 0.441, 95%CI: 0.081–0.801, *P* = 0.016) in the FinnGen Biobank. No association between genetically predicted diverticular disease and colorectal cancer (beta = 0.208, 95%CI: -0.291,0.532, *P* = 0.207) was found in the UK Biobank, but the effect direction was consistent with that in the FinnGen Biobank (Table 1). Heterogeneity among the SNPs was observed in analyses in the Finngen Biobank and UK Biobank, and MR-Egger test also detected horizontal pleiotropy in analysis in the UK Biobank (P__MR−Egger_ intercept = 0.019) (Table 1 and Fig. 1). However, all these three sensitivity methods still showed concordant results in the two databases. In addition, although the MR-PRESSO approach detected one outlier in analysis in the FinnGen Biobank, the association pattern still not changed after removal of the outlier (beta = 0.337, 95%CI: 0.020–0.653, *P* = 0.044) (Table 2). There was little evidence of weak instrument bias.

**Table 1:** Results of bidirectional Mendelian randomization analysis, stratified analysis, and sensitivity analysis

exposure	outcome	outcome data sources	method	N_snp	beta	LCI	UCI	P_− effect_	P_− heterogeneity_	P_− intercept_
diverticular disease	colon cancer	FinnGen	MR Egger	34	0.913	-0.502	2.328	0.215	0.004	0.538
			Weighted median	34	0.690	0.103	1.278	0.021		
			Inverse variance weighted	34	0.489	0.020	0.959	0.041		
		UK Biobank	MR Egger	39	0.989	-0.045	2.023	0.069	0.123	0.125
			Weighted median	39	0.326	-0.145	0.798	0.175		
			Inverse variance weighted	39	0.206	-0.140	0.551	0.243		
	rectum cancer	FinnGen	MR Egger	34	1.066	-0.271	2.402	0.128	0.880	0.259
			Weighted median	34	0.350	-0.291	0.990	0.284		
			Inverse variance weighted	34	0.328	-0.119	0.775	0.151		
		UK Biobank	MR Egger	39	1.723	0.270	3.176	0.026	< 0.001	0.029
			Weighted median	39	0.415	-0.186	1.015	0.176		
			Inverse variance weighted	39	0.134	-0.367	0.636	0.600		
	colorectal cancer	FinnGen	MR Egger	34	0.768	-0.317	1.853	0.175	0.008	0.535
			Weighted median	34	0.561	0.109	1.014	0.015		
			Inverse variance weighted	34	0.441	0.081	0.801	0.016		
		UK Biobank	MR Egger	39	1.309	0.381	2.237	0.009	0.008	0.019
			Weighted median	39	0.120	-0.291	0.532	0.567		
			Inverse variance weighted	39	0.208	-0.115	0.532	0.207		
colorectal cancer	diverticular disease	Meta-analysis of GWASs	MR Egger	64	0.005	-0.004	0.014	0.291	3.80E-06	0.617
			Weighted median	64	0.008	0.005	0.011	4.98E-07		
			Inverse variance weighted	64	0.007	0.004	0.010	4.01E-07		

N_snp, the number of SNPs used in the analysis. LCI, lower 95% confidence interval. UCI, upper 95% confidence interval. P_− heterogeneity,_ the p value of Cochrane’s Q test for inverse variance weighted approach. P_− intercept_, the p value of test for directional pleiotropy by Egger-intercept

**Table 2:** MR-PRESSO results of bidirectional Mendelian randomization analysis

exposure	outcome data sources	outcome	N_snp	outlier	P__Global test_	P__Distortion test_	MR analysis	Beta	LCI	UCI	P
Diverticular disease	FinnGen	Colorectal cancer	34	1	0.009	0.551	Raw	0.409	0.063	0.754	0.026
							Outlier-corrected	0.337	0.020	0.653	0.044
		Colon cancer	34	2	0.003	0.855	Raw	0.412	-0.035	0.860	0.079
							Outlier-corrected	0.447	0.049	0.844	0.034
		Rectum cancer	34	0	0.901	NA	Raw	0.307	-0.052	0.667	0.102
							Outlier-corrected	NA	NA	NA	NA
	UK Biobank	Colorectal cancer	39	0	0.009	NA	Raw	0.153	-0.147	0.452	0.324
							Outlier-corrected	NA	NA	NA	NA
		Colon cancer	39	0	0.159	NA	Raw	0.158	-0.161	0.476	0.337
							Outlier-corrected	NA	NA	NA	NA
		Rectum cancer	39	1	< 0.001	0.041	Raw	0.109	-0.353	0.570	0.647
							Outlier-corrected	-0.017	-0.455	0.422	0.941
Colorectal cancer	Meta-analysis of GWAS	Diverticular disease	64	1	< 0.001	0.709	Raw	0.008	0.006	0.011	1.95E-08
							Outlier-corrected	0.009	0.007	0.011	9.56E-11

N_snp, the number of SNPs used in the analysis. LCI, lower 95% confidence interval. UCI, upper 95% confidence interval. P__Global test,_ the p value of global test in MR-PRESSO analysis. P__Distortion test,_ the p value of distortionl test in MR-PRESSO analysis

In stratified analysis, we found that genetically determined diverticular disease was associated with a higher risk of colon cancer (beta = 0.489, 95%CI: 0.020–0.959, *P* = 0.041) rather than rectum cancer (beta = 0.328, 95%CI: -0.119-0.775, *P* = 0.151) in the FinnGen consortia (Table 1). However, no association was observed between diverticular disease and colon/rectum cancer in the UK Biobank (Table 1). Although moderate heterogeneity and horizontal pleiotropy were found in a part of the analyses, the sensitivity analyses displayed a similar association pattern with the primary analysis (Table 1 and Fig. 1). Similarly, outliers were also detected by using the MR-PRESSO method in a part of the analyses, but the results have not changed substantially.

By using the estimates from the replication stage of the original GWAS for diverticular disease, we generated similar results as if using the discovery stage estimates, that is there was no association between the genetically determined diverticular disease and colorectal/colon/rectum cancer risk in the UK Biobank (Supplementary Table 15). Sensitivity analyses also supported the null association of the diverticular disease with colorectal/colon/rectum cancer (Supplementary Tables 15 and 16).

When applying colorectal cancer as exposure variable and diverticular disease as outcome, we found that genetically predicted colorectal cancer risk was associated with a slightly increased risk of diverticular disease (beta = 0.007, 95%CI: 0.004–0.010, *P* < 0.001). Although obvious heterogeneity among the used SNPs was identified, the results remained consistent with the primary analysis in the sensitivity analyses (Table 1 and Fig. 1). Furthermore, one outlier was detected but no directional change was found after removal of the outlier in the estimates of MR-PRESSO analysis (Table 2).

## Discussion

The present MR study found that genetic predisposition to diverticular disease was associated with the increased risks of colorectal cancer and colon cancer in the FinnGen population. In line with our findings, a population-based and matched cohort study which included 389,184 participants found that patients with diverticular disease had an increased risk of colon cancer and the colorectal cancer risk increased mainly in the first year of follow-up [22]. A systematic review and meta-analysis of observational studies demonstrated the pool prevalence of colorectal cancer was 1.9% in the patients with acute diverticulitis and the risk of colorectal cancer was significantly higher in the patients with complicated diverticulitis [23]. Several studies also suggested an increased risk of colon/colorectal cancer in diverticular disease patients compared to those without the disease [2–5]. However, a population-based case-control study conducted in Sweden did not support a long-term positive association between diverticular disease and colorectal cancer [24], and a nationwide population-based study including 41,359 Taiwan individuals also indicated that diverticular disease was not associated with increased colorectal cancer risk after the first year of follow-up [25]. They attributed the increased risk of colorectal cancer within one year of diagnosis of diverticular disease to surveillance, misclassification, and screening effects [24, 25]. Contrary to our findings, a cross-sectional, prospective study conducted in the Netherlands found a negative association between diverticulosis and colorectal cancer [26]. This conflicting finding may be due to the inherent limitation (for example, residual confounding) of the cross-sectional study design. Furthermore, a phenome-wide association study including 334,385 unrelated White British individuals from the UK Biobank showed that genetic predisposition to colorectal cancer was associated with the risk of diverticular disease, and they also observed a causal association between colorectal cancer and diverticular disease in the follow-up bidirectional MR analysis [27]. These suggested a possible shared aetiology between the two diseases [27]. In addition, the shared risk factors between diverticular disease and colorectal cancer may also be a potential bias [28].

Several potential mechanisms may explain the positive association between diverticular disease and colorectal cancer. Dietary factors were found to be involved in both pathogeneses of diverticular disease and colorectal cancer, especially low fiber intake [29, 30]. Low fiber diet causes excessive segmental contraction of the colon, which further increases intraluminal pressure, promotes mucosal herniation, and facilitates diverticulum formation [31]. Besides, Dietary fiber deficiency can lead to an increment of abnormal movement of the colon along with aging, which results from abnormal thickening of the muscles in the colonic wall [31]. The contact time between carcinogens and the luminal epithelium would also increase due to decreased fecal bulk, concentrated carcinogens, and slowed transit [32, 33], which is thought beneficial to the development of colorectal cancer. Cellular proliferation dysregulation may link diverticular disease to colorectal cancer. It causes oxidative stress and genetic alterations among cells of inflamed colonic mucosa before the histologic changes and therefore is thought to be the earliest event of colorectal carcinoma [34, 35]. Compared with healthy controls, an upregulation of cellular proliferation of colonic mucosa was found among the patients with diverticular disease [36, 37]. Moreover, certain anaerobic bacteria produce bile acid derivatives which may accumulate in the diverticulum and play the role of co-carcinogens [38, 39].

There are several strengths of the present study. The major one is the MR design, which has a higher level of evidence and can compensate for some of the limitations inherent in observational studies, such as reverse causation and residual confounding. In addition, we examined the association in two datasets, the UK Biobank and the FinnGen Biobank, and the consistent effect direction boosted the reliance of our results. The analyses were confined to participants of two populations, the Finngen Biobank and the UK Biobank, which thus minimized the population structure bias. This confinement on the other side limits the generalizability of our findings to other populations.

Limitations need to be considered when interpreting our results. The first one is that there is a sample overlap in the analysis of the UK Biobank which would introduce bias in the MR estimates in the direction of the observational study. Therefore, we employed the betas from the replication analysis in the original GWAS to re-analysis and compared the estimates with the original results. We found little change in the results, which indicated that the sample overlap incurred a small bias in the UK Biobank analysis. However, we also found a sample overlap in the MR analysis when using colorectal cancer as exposome and diverticular disease as outcome. Although the findings indicated a potential association between colorectal cancer and diverticular disease, it should be interpreted with caution and needs to be verified in further MR studies. Another limitation that needs to be considered is the winner’s curse in the MR analysis. Since the strong instruments used in this study, perhaps this bias is not very large. Horizontal pleiotropy also challenges causal inference in any MR study. However, the consistent results from all sensitivity analyses indicated that the influence of horizontal pleiotropy may be small in this study. Finally, diverticular disease is a binary exposure, and as such the results cannot be interpreted in the same way as if using a continuous exposure, but have to be interpreted with caution.

## Conclusion

In conclusion, this MR study suggests genetically determined diverticular disease may be a risk factor for colorectal and colon cancer in the FinnGen population. However, the effect of colorectal cancer on diverticular disease needs to be further verified.

## Electronic supplementary material

Below is the link to the electronic supplementary material.

Supplementary Material 1

Supplementary Material 2

Supplementary Material 3

## Data Availability

The summary-level data used in this study can be obtained from two publicly available datasets, the UK Biobank (https://docs.google.com/spreadsheets/d/1AeeADtT0U1AukliiNyiVzVRdLYPkTbruQSk38DeutU8/edit#gid=268241601) and the FinnGen Biobank (https://storage.googleapis.com/finngen-public-data-r8/summary_stats/R8_manifest.tsv). One can find these summary-level data according to the navigation on the corresponding official websites. The summary-level data download links were displayed in Supplementary Table 4. In addition, the data used in this study were presented in Supplementary Tables 5–14, and the code for MR analysis was provided in Supplementary File 1.

## List of abbreviations

MR: Mendelian randomization.
SNPs: single nucleotide polymorphisms.
