# Supplementary material for: Association between diverticular disease and colorectal cancer: a bidirectional mendelian randomization study
Source: BMC Cancer. 2023 Feb 10;23:137. doi: 10.1186/s12885-023-10606-x (PMC9912649; doi:10.1186/s12885-023-10606-x)
Supplement: Supplementary file 1 — Supplementary Material 1 [file 12885_2023_10606_MOESM1_ESM.docx]

library(TwoSampleMR)

exp_dat <- read_exposure_data(

filename = "CRC_DD.csv",

sep = ",",

snp_col = "SNP",

beta_col = "beta.exposure",

se_col = "se.exposure",

effect_allele_col = "effect_allele.exposure",

other_allele_col = "other_allele.exposure",

eaf_col = "eaf.exposure",

pval_col = "pval.exposure",

)

#exp<-clump_data(exp_dat,clump_r2 = 0.01)

#write.table(exp,file="D:\\MR\\crc_0.01clumping.csv",sep=",",row.names = F)

outcome_dat <- read_outcome_data(

snps = exp_dat$SNP,

filename = "CRC_DD.csv",

sep = ",",

snp_col = "SNP",

beta_col = "BETA",

se_col = "SE",

eaf_col = "A1FREQ",

effect_allele_col = "ALLELE1",

other_allele_col = "ALLELE0",

pval_col = "P",

)

dat <- harmonise_data(

exposure_dat = exp_dat,

outcome_dat = outcome_dat,

action=3

)

res <- mr(dat)

write.table(res,file="CRC_DD_results.csv",row.names=FALSE,col.names=TRUE,sep=",")

res_heterogeneity=mr_heterogeneity(dat)

res_pleiotropy=mr_pleiotropy_test(dat)

res_single <- mr_singlesnp(dat)

tiff(file="D:\\update analysis\\figure1.tiff",units='cm',width=10,height=10,res=300,

compression = "lzw")

p1 <- mr_scatter_plot(res, dat)

p1[[1]]

dev.off()

tiff(file="D:\\update analysis\\figure2.tiff",units='cm',width=12,height=15,res=300,

compression = "lzw")

p2 <- mr_forest_plot(res_single)

p2[[1]]

dev.off()

save(dat, res, res_heterogeneity, res_pleiotropy, res_single, file="CRC_DD.Rdata")

mr_report(dat)

#MR PRESSO#

library(MRPRESSO)

mr_presso(BetaOutcome = "beta.outcome", BetaExposure = "beta.exposure", SdOutcome = "se.outcome", SdExposure = "se.exposure",

OUTLIERtest = TRUE, DISTORTIONtest = TRUE, data = dat, NbDistribution = 1500, SignifThreshold = 0.05)
