# Supplementary material for: Association between diverticular disease and colorectal cancer: a bidirectional mendelian randomization study
Source: BMC Cancer. 2023 Feb 10;23:137. doi: 10.1186/s12885-023-10606-x (PMC9912649; doi:10.1186/s12885-023-10606-x)
Supplement: Supplementary file 2 — Supplementary Material 2 [file 12885_2023_10606_MOESM2_ESM.pdf]

## STROBE-MR checklist of recommended items to address in reports of Mendelian randomization studies[1]

| Item No. | Section | | Checklist item | Page No. | Relevant text from manuscript |
|---|---|---|---|---|---|
| 1 | **TITLE and ABSTRACT** | | Indicate Mendelian randomization (MR) as the study's design in the title and/or the abstract if that is a main purpose of the study | 1-2 | Association between diverticular disease and colorectal cancer: a bidirectional Mendelian randomization study |
| | **INTRODUCTION** | | | | |
| 2 | **Background** | | Explain the scientific background and rationale for the reported study. What is the exposure? Is a potential causal relationship between exposure and outcome plausible? Justify why MR is a helpful method to address the study question | 3 | Diverticular disease is a common digistive tract disease and has been associated with colorectal cancer ... MR analysis can strengthen the causal inference in exposure-outcome associations by minimizing residual confounding and reverse causality. |
| 3 | **Objectives** | | State specific objectives clearly, including pre-specified causal hypotheses (if any). State that MR is a method that, under specific assumptions, intends to estimate causal effects | 3 | we conducted a bidirectional MR study to examine the potential causal association between diverticular disease and colorectal cancer. |
| | **METHODS** | | | | |
| 4 | **Study design and data sources** | | Present key elements of the study design early in the article. Consider including a table listing sources of data for all phases of the study. For each data source contributing to the analysis, describe the following: | 3-4,5 | To evaluate the association between diverticular disease and colorectal cancer, we performed a bidirectional MR study...In addition, stratified analysis by site-specific cancer was performed to evaluate the association between diverticular disease and colon/rectum cancer risk with the summary-level data obtained from the FinnGen and UK Biobank. The data download link was provided in **Supplementary Table 4**. |
| | | a) | Setting: Describe the study design and the underlying population, if possible. Describe the setting, locations, and relevant dates, including periods of recruitment, exposure, follow-up, and data collection, when available. | 4-5 | Fifty-one single nucleotide polymorphisms (SNPs) associated with diverticular disease at the genome-wide significance threshold...Details of the replication sample were described elsewhere. SNPs of colorectal cancer at the genome-wide association ...95 variants that were associated with colorectal cancer. |
| | | b) | Participants: Give the eligibility criteria, and the sources and methods of selection of participants. Report the sample size, and whether any power or sample size calculations were carried out prior to the main analysis | 4-5 | |
| | | c) | Describe measurement, quality control and selection of genetic variants | 4-5 | Linkage disequilibrium among the remaining 48 SNPs was estimated...can explain a 1.26% variance for diverticular disease. we evaluated LD among these SNPs ...can be explained by the used instruments was 16.97% |
| | | d) | For each exposure, outcome, and other relevant variables, describe methods of assessment and diagnostic criteria for diseases | 4-5 | Details of the replication sample were described elsewhere. The data download link was provided in **Supplementary Table 4**. |
| | | e) | Provide details of ethics committee approval and participant informed consent, if relevant | 5 | Ethics committee approval and participant informed consent were obtained by each study |
| 5 | **Assumptions** | | Explicitly state the three core IV assumptions for the main analysis (relevance, independence and exclusion restriction) as well assumptions for any additional or sensitivity analysis | 5-6 | Three main assumption should be considered when conducting MR analysis: (1 instrumental variables are strongly correlated with exposures of interest; (2) instruments are not related to the potential confounders; (3) the selected geneti variants should affect the outcome only via the exposures of interest. |
| 6 | **Statistical methods: main analysis** | | Describe statistical methods and statistics used | 5-6 | The random effect inverse-variance weighted MR method was used as the primary method, and MR estimates were performed in beta values because the exposure and outcome are all binary variables. Several sensitivity analyses, including the weighted median [17], MR-Egger [18], and MR-PRESSO [19] methods, were conducted to examine the consistency of results and to detect horizontal pleiotropy. |

| | | | | |
|---|---|---|---|---|
| | | a) Describe how quantitative variables were handled in the analyses (i.e., scale, units, model) | none | |
| | | b) Describe how genetic variants were handled in the analyses and, if applicable, how their weights were selected | 4-5 | The random effect inverse-variance weighted MR method was used as the primary method, and MR estimates were performed in beta values because the exposure and outcome are all binary variables |
| | | c) Describe the MR estimator (e.g. two-stage least squares, Wald ratio) and related statistics. Detail the included covariates and, in case of two-sample MR, whether the same covariate set was used for adjustment in the two samples | 5-6 | The random effect inverse-variance weighted MR method was used as the primary method...Besides, Cochrane's Q test was used to assess the heterogeneity among estimates of SNPs in one analysis. |
| | | d) Explain how missing data were addressed | none | |
| | | e) If applicable, indicate how multiple testing was addressed | none | |
| 7 | **Assessment of assumptions** | Describe any methods or prior knowledge used to assess the assumptions or justify their validity | 5-6 | Three main assumption should be considered when conducting MR analysis [16]: (1) instrumental variables are strongly correlated with exposures of interest; (2) instruments are not related to the potential confounders; (3) the selected genetic variants should affect the outcome only via the exposures of interest. |
| 8 | **Sensitivity analyses and additional analyses** | Describe any sensitivity analyses or additional analyses performed (e.g. comparison of effect estimates from different approaches, independent replication, bias analytic techniques, validation of instruments, simulations) | 5-6 | Several sensitivity analyses, including the weighted median [17], MR-Egger [18], and MR-PRESSO [19] methods, were conducted to examine the consistency of results and to detect horizontal pleiotropy...Besides, Cochrane's Q test was used to assess the heterogeneity among estimates of SNPs in one analysis. |
| 9 | **Software and pre-registration** | | | |
| | | a) Name statistical software and package(s), including version and settings used | 6 | All tests were two-sided and performed using the "TwoSampleMR" (version:0.5.6) [20] and "MR-PRESSO" (version: 1.0) [19] packages in the R software (version 4.1.3). |
| | | b) State whether the study protocol and details were pre-registered (as well as when and where) | none | |
| | **RESULTS** | | | |
| 10 | **Descriptive data** | | | |
| | | a) Report the numbers of individuals at each stage of included studies and reasons for exclusion. Consider use of a flow diagram | 3-5 | which included 31,964 cases and 419,135 controls coming from the UK Biobank. a combined European sample of 3,893 cases and 2,829 controls was included. a combined meta-analysis of GWASs with a sample of 125,478 individuals. which was conducted among 31,964 cases and 419,135 controls in the UK Biobank |
| | | b) Report summary statistics for phenotypic exposure(s), outcome(s), and other relevant variables (e.g. means, SDs, proportions) | 3-5 | Summary-level data on associations of diverticular disease-associated SNPs with colon cancer, rectum cancer, and colorectal cancer were obtained from the FinnGen consortium [14] and the UK Biobank study. The effect estimates of colorectal cancer-associated SNPs on diverticular disease were derived from a publicly available meta-analysis of GWASs. |
| | | c) If the data sources include meta-analyses of previous studies, provide the assessments of heterogeneity across these studies | none | |
| | | d) For two-sample MR:<br>i. Provide justification of the similarity of the genetic variant-exposure associations between the exposure and outcome samples | 5 | All the estimates used in the MR analyses were displayed in **Supplementary Table 5-14**. |

| | | | | | |
|---|---|---|---|---|---|
| | | | ii. Provide information on the number of individuals who overlap between the exposure and outcome studies | 4, 8-9 | Due to the sample overlap between the exposure and outcome in the UK Biobank, we also employed the beta and se from the replication stage '[12] of the original GWAS for diverticular disease to validate our primary findings in the UK Biobank (**Supplementary Table 2**). |
| 11 | **Main results** | | | | |
| | | a) | Report the associations between genetic variant and exposure, and between genetic variant and outcome, preferably on an interpretable scale | 5 | All the estimates used in the MR analyses were displayed in **Supplementary Table 5-14**. |
| | | b) | Report MR estimates of the relationship between exposure and outcome, and the measures of uncertainty from the MR analysis, on an interpretable scale, such as odds ratio or relative risk per SD difference | 6-7 | Genetic predisposition to diverticular disease was associated with increased risks of colorectal cancer (beta=0.441, 95%CI: 0.081-0.801, *P*=0.016)... |
| | | c) | If relevant, consider translating estimates of relative risk into absolute risk for a meaningful time period | none | |
| | | d) | Consider plots to visualize results (e.g. forest plot, scatterplot of associations between genetic variants and outcome versus between genetic variants and exposure) | Figure 1 | Figure 1. Scatter plots of Mendelian randomization analysis |
| 12 | **Assessment of assumptions** | | | | |
| | | a) | Report the assessment of the validity of the assumptions | 6-7 | Heterogeneity among the SNPs was observed in analyses in the Finngen Biobank and UK Biobank, and MR-Egger test also detected horizontal pleiotropy in analysis in the UK Biobank...There was little evidence of weak instrument bias. |
| | | b) | Report any additional statistics (e.g., assessments of heterogeneity across genetic variants, such as $I^2$, Q statistic or E-value) | 6-7 | |
| 13 | **Sensitivity analyses and additional analyses** | | | | |
| | | a) | Report any sensitivity analyses to assess the robustness of the main results to violations of the assumptions | 6-7 | Heterogeneity among the SNPs was observed in analyses in the Finngen Biobank and UK Biobank, and MR-Egger test also detected horizontal pleiotropy in analysis in the UK Biobank...There was little evidence of weak instrument bias. |
| | | b) | Report results from other sensitivity analyses or additional analyses | 6-7 | In stratified analysis, we found that genetically determined diverticular disease was associated with a higher risk of colon cancer... |
| | | c) | Report any assessment of direction of causal relationship (e.g., bidirectional MR) | 6-7 | When applying colorectal cancer as exposure variable and diverticular disease as outcome, we found that genetically predicted colorectal cancer risk was associated with a slightly increased risk of diverticular disease |
| | | d) | When relevant, report and compare with estimates from non-MR analyses | none | |
| | | e) | Consider additional plots to visualize results (e.g., leave-one-out analyses) | none | |
| | **DISCUSSION** | | | | |
| 14 | **Key results** | | Summarize key results with reference to study objectives | 7-8 | The present MR study found that genetic predisposition to diverticular disease was associated with the increased risks of colorectal cancer and colon cancer in the FinnGen population. |
| 15 | **Limitations** | | Discuss limitations of the study, taking into account the validity of the IV assumptions, other sources of potential bias, and imprecision. Discuss both direction and magnitude of any potential bias and any efforts to address them | 8-9 | Limitations need to be considered when interpreting our results. The first one is that there is a sample overlap in the analysis of the UK Biobank which would introduce bias in the MR estimates in the direction of the observational study. |

| | | | | | |
|---|---|---|---|---|---|
| 16 | **Interpretation** | | | | In line with our findings, a population-based and matched cohort study which included 389,184 participants found that patients with diverticular disease had an increased risk of colon cancer and the colorectal cancer risk increased mainly in the first year of follow-up... |
| | | a) | Meaning: Give a cautious overall interpretation of results in the context of their limitations and in comparison with other studies | 7-8 | |
| | | b) | Mechanism: Discuss underlying biological mechanisms that could drive a potential causal relationship between the investigated exposure and the outcome, and whether the gene-environment equivalence assumption is reasonable. Use causal language carefully, clarifying that IV estimates may provide causal effects only under certain assumptions | 8 | Several potential mechanisms may explain the positive association between diverticular disease and colorectal cancer.Dietary factors were found to be involved in both pathogeneses of diverticular disease and colorectal cancer, especially low fiber intake... |
| | | c) | Clinical relevance: Discuss whether the results have clinical or public policy relevance, and to what extent they inform effect sizes of possible interventions | none | |
| 17 | **Generalizability** | | Discuss the generalizability of the study results (a) to other populations, (b) across other exposure periods/timings, and (c) across other levels of exposure | 8 | The confinement on the other side limits the generalizability of our findings to other populations. |
| | **OTHER INFORMATION** | | | | |
| 18 | **Funding** | | Describe sources of funding and the role of funders in the present study and, if applicable, sources of funding for the databases and original study or studies on which the present study is based | 10 | The authors declare that no funds, grants, or other support were received during the preparation of this project. |
| 19 | **Data and data sharing** | | Provide the data used to perform all analyses or report where and how the data can be accessed, and reference these sources in the article. Provide the statistical code needed to reproduce the results in the article, or report whether the code is publicly accessible and if so, where | 10 | The summary-level data download links were displayed in **Supplementary Table 4**. In addition, the data used in this study were presented in **Supplementary Tables 5-14**, and the code for MR analysis was provided in **Supplementary File 1**. |
| 20 | **Conflicts of Interest** | | All authors should declare all potential conflicts of interest | 10 | All authurs declare no competing interest. |

This checklist is copyrighted by the Equator Network under the Creative Commons Attribution 3.0 Unported (CC BY 3.0) license.

1. Skrivankova VW, Richmond RC, Woolf BAR, et al. Strengthening the Reporting of Observational Studies in Epidemiology using Mendelian Randomisation (STROBE-MR): Explanation and Elaboration. BMJ. 2021;375:n2233.
